# Interaction of dermatologically relevant nanoparticles with skin cells and skin

**DOI:** 10.3762/bjnano.5.245

**Published:** 2014-12-08

**Authors:** Annika Vogt, Fiorenza Rancan, Sebastian Ahlberg, Berouz Nazemi, Chun Sik Choe, Maxim E Darvin, Sabrina Hadam, Ulrike Blume-Peytavi, Kateryna Loza, Jörg Diendorf, Matthias Epple, Christina Graf, Eckart Rühl, Martina C Meinke, Jürgen Lademann

**Affiliations:** 1 Department of Dermatology and Allergy, Charité-Universitaetsmedizin Berlin, Chariteplatz 1, 10117 Berlin, Germany; 2 Kim Il Sung University, Ryongnam-Dong, Taesong District, Pyongyang, DPR Korea; 3 Inorganic Chemistry and Center for Nanointegration Duisburg-Essen (CeNIDE), University of Duisburg-Essen, Universitaetsstr. 5-7, 45117 Essen, Germany; 4 Physical and Theoretical Chemistry, Freie Universitaet Berlin, Takustr. 3, 14195 Berlin, Germany

**Keywords:** hair follicle, nanodermatology, nanoparticle penetration, skin barrier

## Abstract

The investigation of nanoparticle interactions with tissues is complex. High levels of standardization, ideally testing of different material types in the same biological model, and combinations of sensitive imaging and detection methods are required. Here, we present our studies on nanoparticle interactions with skin, skin cells, and biological media. Silica, titanium dioxide and silver particles were chosen as representative examples for different types of skin exposure to nanomaterials, e.g., unintended environmental exposure (silica) versus intended exposure through application of sunscreen (titanium dioxide) or antiseptics (silver). Because each particle type exhibits specific physicochemical properties, we were able to apply different combinations of methods to examine skin penetration and cellular uptake, including optical microscopy, electron microscopy, X-ray microscopy on cells and tissue sections, flow cytometry of isolated skin cells as well as Raman microscopy on whole tissue blocks. In order to assess the biological relevance of such findings, cell viability and free radical production were monitored on cells and in whole tissue samples. The combination of technologies and the joint discussion of results enabled us to look at nanoparticle–skin interactions and the biological relevance of our findings from different angles.

## Introduction

The skin is the outermost surface of humans and therefore easily accessible. The exposure of skin to nanomaterials can be categorized into unintended exposure to engineered particles and intended exposure, which includes compounds meant to stay on the skin surface (sunscreens, antiseptics) or those meant to enter viable skin (dermatotherapy, cosmetics), respectively. With the increasing use of nanoscale architectures in all of these fields, the question as to whether a nanomaterial deposited on the skin surface is capable of penetrating horny layers and reaching viable epidermis is of high relevance.

As a result of the special architecture of the skin, levels of interactions include the translocation step across the skin barrier, cellular uptake as well as biological effects. In fact, biological responses to nanoparticle exposure may occur on the cellular level, but also as a result of interactions with the skin microenvironment. In the following, we present results obtained from own studies on the interactions of skin, skin cells and biological media with silica, titanium dioxide and silver particles as representatives for nanomaterials of high relevance from the dermatological perspective.

## Results and Discussion

### Skin barrier translocation of nanomaterials

**Figure 1:**
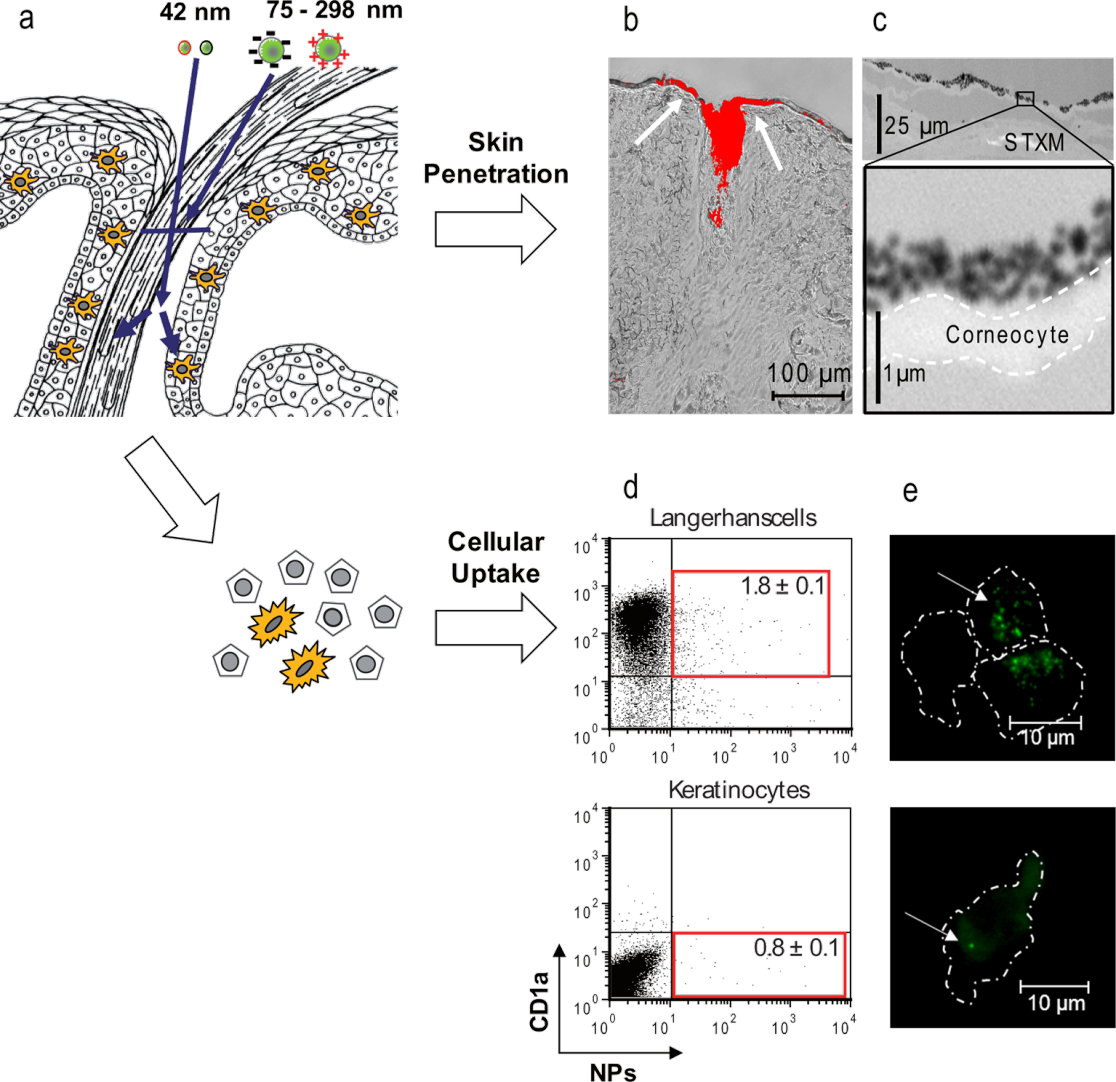
Interdisciplinary set-up to study skin penetration and cellular uptake of amorphous silica particles in human skin explants. Silica particles with 42, 75 or 300 nm diameter were applied on excised human skin to study penetration and cellular uptake (a). Labeling of particles with fluorescein enabled the visualization of particle accumulation on skin sections and in hair follicle openings by using fluorescence microscopy (b). However, single particles on the skin surface could only be visualized after preparation of silica particles with gold cores and skin section analysis by using scanning transmission X-ray microscopy (STXM) (c). Whereas particles with a size ranging between 75 and 300 nm accumulated on the horny layers and in hair follicle openings without deeper penetration, flow cytometry of single cell suspensions prepared from skin tissue pretreated with fluorescent 42 nm particles identified a small percentage of cells associated with particles (d, boxed areas in representative flow cytometry images). Single cell fluorescence microscopy confirmed the presence of cell-associated particles that are highly suggestive for cellular uptake (e). (Figure 1a,b,d,e modified with permission from [3], Copyright 2012 American Chemical Society; Figure 1c modified with permission from [4], Copyright 2009 Society of Photo Optical Instrumentation Engineers.)

The first contact of nanomaterial occurs with the horny layers of terminally differentiated corneocytes. Pathways across the intact stratum corneum have been postulated for some, mostly deformable, particles, such as liposomes or transferosomes. [1–2]. Although increasing reports suggest that barrier translocation of solid particles occurs especially when the skin barrier is disrupted, the penetration of solid particles into the viable epidermis seems to be limited. Figure 1 illustrates the experimental set-up that we chose in order to investigate skin penetration of topically applied silica particles (Figure 1a).

Here, conventional fluorescence microscopy of skin sections yielded no evidence for the penetration of 42–300 nm fluorescent silica particles in excised human skin. The data are in accordance with differential tape stripping studies by our group [5], which show that for different particle architectures, approx. 95% of the applied particles remain in the upper layers of the stratum corneum. Because conventional optical microscopy faces clear limitations due to the lack of spatial resolution, we conducted scanning transmission X-ray microscopy (STXM) studies on human skin, which allowed us to visualize silica-shell/gold-core particles in the size range of 94–298 nm on superficial layers of the stratum corneum and in hair follicle openings at the single particle level (Figure 1c, see [4] for further details). Our results are in line with many other studies on particles that are in this size range and larger. For example, in the case of titanium dioxide particles, a deeper penetration was not detected through microscopy, both for microparticles as well as for nanoparticulate preparations [6–8]. To generate valid data, however, it is not sufficient to rely on the penetration depth alone [9]. A deeper understanding can only be obtained by combining different approaches. Notably, X-ray microscopy could become a valuable tool for imaging with high spatial resolution combined with analysis of spectroscopic data. Following similar approaches, Adachi et al. performed transmission electron microscopy (TEM) and energy dispersive X-ray spectroscopy (EDX) measurements on murine skin after up to 8 weeks of daily sunscreen application with similar results [10]. However, a loss of particles during fixation and sectioning poses technical challenges, especially when ultrathin sections must be prepared for analysis with high resolution techniques, such as STXM or electron microscopy. Preparation of single-cell suspensions from tissue samples pretreated with nanoparticles overcomes challenges associated with fixation and sectioning. The cells remain intact and can be analyzed by flow cytometry or single cell microscopy.

For our studies on skin penetration of silica particles, we prepared single-cell suspensions of skin samples treated with fluorescent particles and performed flow cytometry and single-cell microscopy on keratinocytes, Langerhans cells as well as dermis cell isolates. Although deeper penetration through the horny layers into the viable epidermis could not or could only partially be observed even after mild skin barrier disruption by means of cyanoacrylate skin surface stripping, we were able to isolate skin cells which had taken up particles from treated ex vivo human skin (Figure 1d) [3,11]. In accordance with previous studies, the particle size appeared to be a major determinant for cellular uptake. Notably, after ex vivo topical application of silica particles on human skin and subsequent isolation of keratinocytes and Langerhans cells, only the internalization of 42 nm, but not of 75 or 200 nm particles could be identified. Interestingly, the size limit for penetration and cellular uptake appears to differ among different particle types. In previous studies of our groups, we observed penetration and cellular uptake of fluorescent polystyrene particles ranging from 40–200 nm in diameter after skin surface stripping in murine and human skin [11–12]. Furthermore, the internalization of a fluorescent vaccinia virus vector (diameter approx. 290 nm) could be conveniently identified not only in murine hair follicle epithelium [12], but also in dendritic cells of the skin. In fact, our earlier studies and results of others suggest that even low penetration rates of particle-bound antigens may result in cellular uptake by cutaneous antigen-presenting cells and relevant immune response [12–14]. Furthermore, low penetration rates may become relevant, when large skin surface areas come in contact with the respective nanomaterial, or when repetitive exposure occurs over prolonged time periods.

The susceptibility to artefacts also underlines the value of methods that enable studies on the whole skin, ideally under in vivo conditions, e.g., in vivo confocal microscopy and multiphoton microscopy [9]. In earlier studies using mice, we were able to monitor the penetration of fluorescent 200 nm particles in hair follicles and diffusion into perifollicular tissues in vivo over time [12]. On the other hand, hair follicles were found to be excretion pathways for injected gold nanoparticles [15].

**Figure 2:**
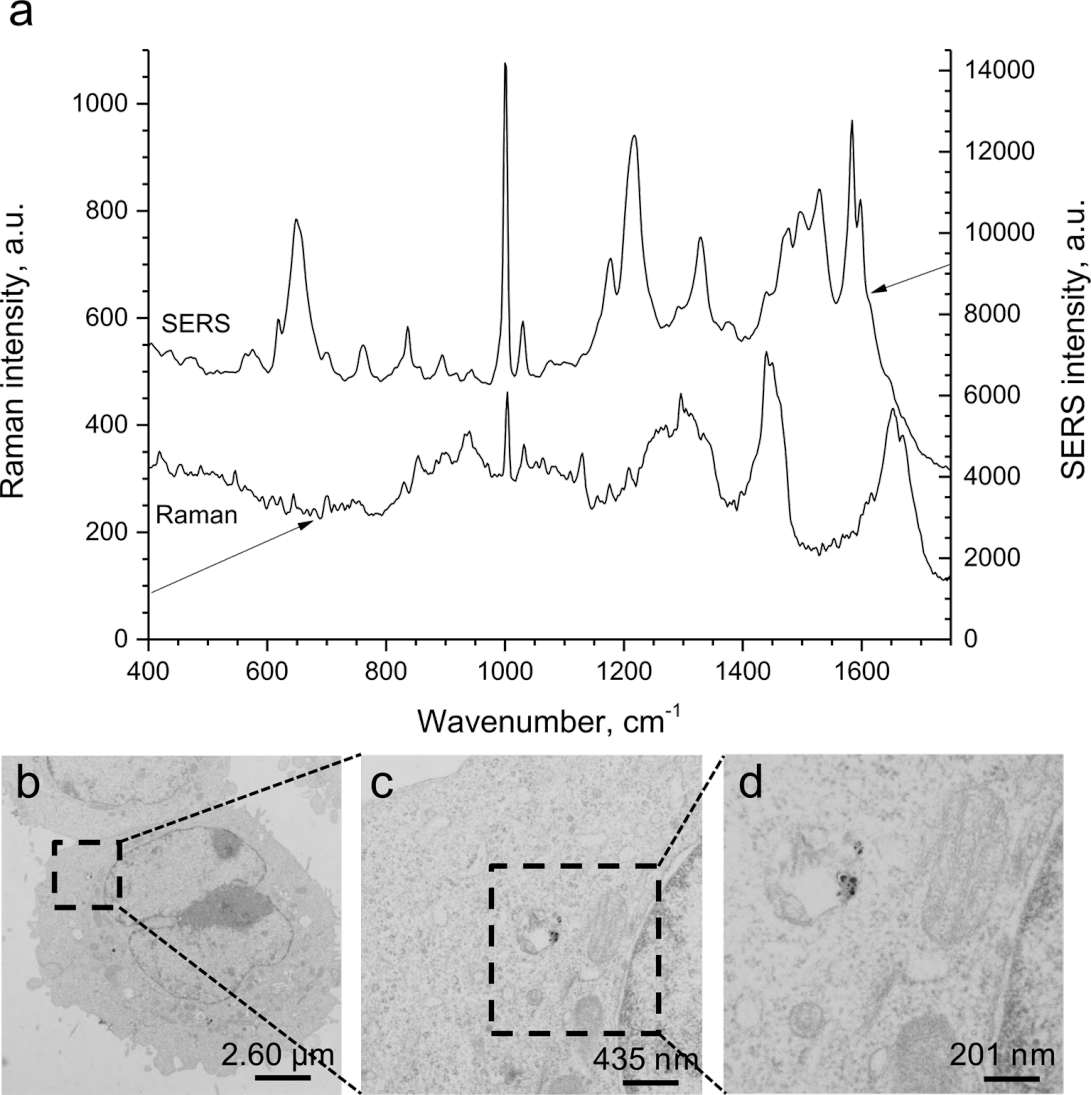
Skin penetration and cellular uptake of silver nanoparticles (AgNP). While studies with silica particles required labeling of particles with fluorescent dyes or introduction of gold cores followed by sectioning of the tissue for further analysis, skin penetration of AgNP could be studied in intact tissue blocks by tracking of the Raman signal. The representative Raman and SERS typical spectra were obtained in intact porcine skin (depth 4 µm) pre-treated with AgNP, excitation wavelength 785 nm, power on the sample 25 mW, analyzing range 400–2000 cm^−1^ (a). Label-free detection of AgNP also facilitated studies on cellular uptake by HaCaT cells by using TEM. In the representative TEM images of HaCaT cells (obtained after incubation for 24 h with 25 µg/mL AgNP) AgNP are accumulated in endosomes (b–d).

Raman microscopy is another technique with high spatial resolution which permits such studies. While we gathered first own results on skin with the in vivo detection of antioxidant levels as indicators of oxidative stress [16], it is now increasingly being used to study particle–skin interactions [17–18]. Yet, not all particle types are equally suited for such investigations. In the following, we report our results on confocal Raman microscopy for analyzing the skin penetration of silver nanoparticles (AgNP, mean size 70 nm) in porcine ear skin. By tracking the Raman signal of AgNP, the mean penetration depth in intact skin was found to be 4.4 ± 1.5 µm, which is in accordance with other investigations on silica [3], zink oxide [19], or AgNP in this size range or smaller [20]. A pre-treatment with tape stripping of 20 adhesive tapes, which according to our own unpublished data corresponds to a removal of approximately 70–80% of the stratum corneum, only slightly increased the penetration depth to 5.1 ± 2.5 µm. Additionally, the penetration profile of AgNP was analyzed by the highly sensitive tracking of the surface enhanced Raman scattering (SERS) signal of single AgNP. Here, the penetration depth was found to be 19 ± 10 µm for intact skin, compared to 22 ± 5 µm for skin pre-treated with 20 tape stripes. This effect is well known for AgNP of this type and size [20]. Results obtained from SERS indicate that single AgNPs can penetrate deeply into the stratum corneum. The Raman and SERS spectra of porcine skin pre-treated with AgNP are shown in Figure 2a.

The results illustrate that for AgNP, the SERS effect can be used to monitor the skin penetration depth of single particles. Interestingly, pretreatment of skin with 20 tape strippings doubled the likelihood to detect a SERS effect. In these tape-stripped skin samples, the SERS effect was measured deep in the stratum corneum and in the stratum granulosum of the viable epidermis. This finding could indicate a deeper penetration in moderately disrupted skin. However, secondary translocation through microscopic injuries or slantingly growing hair follicles has to be excluded. In fact, the reservoir function of hair follicles for large molecules and particles is now widely accepted [21–22]. The extension of the hair follicle canal deep into the dermis can result in dermal signals, which correspond to particles in such follicular depots rather than free particles in dermis. Such preferred agglomeration and even deep penetration into hair follicles as well as the retention over several days [23] have convincingly been shown for many different particle architectures [11,24–25]. Consistently, we also found depot formation after topical application of fluorescent silica particles. Aggregates retained in the hair follicle are protected from regular shedding and are prone to intense interactions with hair follicle epithelium. In studies with polystyrene particles as well as Modified Vaccinia Ankara Virus as an example for biologically and immunologically relevant particulates in the context of transcutaneous vaccination, we recently identified hair follicles as sites of nanomaterial translocation into the viable tissue, especially when mild skin barrier disruption by tape stripping techniques was performed additionally [11–12]. Similarly, penetration of cobalt nanoparticles in the size range of 20–500 nm were found both in intact and abraded human skin [26–28], while Abdel-Mottaleb et al. confirmed particle penetration and accumulation in inflamed skin [29]. Also, combinations of nanomaterial exposure with UV-irradiation, may be especially deleterious for the skin organ because UV-exposure may facilitate penetration [30] as shown by Mortensen et al. for rigid metallic nanoparticles [31]. This can cause particle disintegration leading to a reduction of the size of the particles with an increased likelihood of penetration [32] or it can trigger photocatalytic processes which cause secondary harm to skin cells [33–34].

### Identification of factors which influence cellular uptake

**Figure 3:**
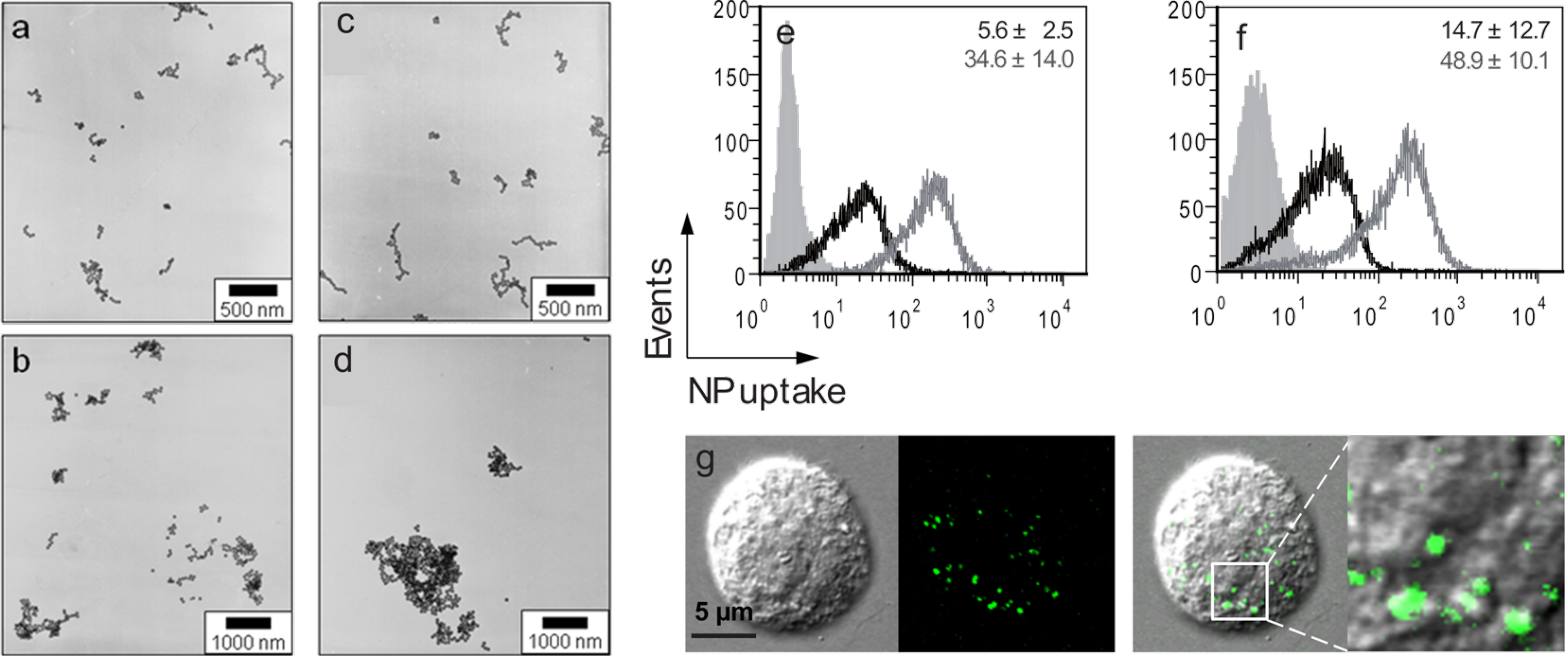
Uptake of fluorescent silica nanoparticles with variable size and surface functionalization by HaCaT cells. Different type of particles were prepared (a–d) with a size of 42 (a,c) or 75 nm (b,d) as well as negative (a,b) or positive (c,d) surface charge through funtionalization with (3-aminopropyl)triethoxysilane (APS) groups. Cells were incubated with particles (10 µg/mL, 2 h, 37 °C) and analyzed by means of flow cytometry (e,f) and confocal laser scanning microscopy (g). Cells incubated with non-functionalized (black lines) and APS-functionalized (grey lines) SiO_2_ particles showed a positive, particle-related signal with respect to the untreated control cells (filled silver histograms). CLSM (Olympus FV1000) confirmed the internalization of both particle aggregates and single particles. Inset shows the four fold magnification of the boxed area. (Modified with permission from [3], Copyright 2012 American Chemical Society.)

In our studies, the functionalization of silica particles with amino groups in order to turn the surface potential of the particles from initially negative to highly positive did not significantly affect cellular uptake rates in whole-tissue experiments. However, immortalized human keratinocytes (HaCaT, Human Adult Low Calcium High Temperature Keratinocytes) and primary human keratinocytes showed an increased uptake of silica particles with positive surface charge under cell culture conditions, which was due to functionalization, e.g., through (3-aminopropyl)triethoxysilane (APS) (Figure 3) [3].

In the case of these silica particles, such a surface functionalization was contrasted by an increased tendency to form aggregates which could explain why barrier translocation did not occur despite an increased cellular uptake. The results demonstrate that although surface functionalization may have some impact on the cellular uptake, particle size and the size of aggregates formed in physiological environments can become limiting factors. Similar results were obtained for similarly sized silica particles (55 ± 2 nm) with and without APS-functionalization in HeLa cells [35]. Also in this case, the APS-functionalized particles were heavily aggregated but still taken up into cells in large numbers. However, *N*-(6-aminohexyl)-aminopropyltrimethoxysilane (AHAPS)-functionalized particles, which had also a highly positive zeta-potential due to the amino groups but did not aggregate in cell culture media were also found in large numbers in the cells [35]. In our studies on the stability of differently functionalized silica particles, even different standard cell culture media compositions resulted in different aggregation behaviors of nanoparticle preparations [35]. The multitude of possible interactions on the skin surface and in the tissue raises the question whether nanomaterials ever have the chance to translocate the skin barrier on the single-particle level, or how the adsorption of skin surface material and secondary changes in particle properties will affect penetration and internalization by cells. Also, results obtained from cell culture conditions are not always predictive for ex vivo or in vivo tissue studies. For example, in previous studies on skin interactions with biodegradable poly(lactic acid) (PLA) particles loaded with different fluorescent dyes, we found that although mono-dispersed and stable in aqueous solution, skin contact with the particles lead to destabilization with the release of loaded dyes [36–37].

The studies further illustrated that cells, especially immortalized cell lines compared to primary cells as well as cell types, e.g., epithelial cells versus dendritic cells, differ significantly in their ability to take up nanomaterial. The choice of the experimental system has a major influence on the generated information and a thorough quality control of the behavior of different particle batches in the experimental models is essential.

### Nanoparticle-induced biological effects in cells and whole skin

Titanium dioxide particles in sunscreens as well as silver particles for skin surface antisepsis are usually intended to stay on the skin surface. Although penetration of solid particles across the intact skin barrier seems to be very limited, experimental data strongly suggests that penetration is enhanced when the skin barrier is disrupted, e.g., after physical, chemical, inflammatory damage, or high UV exposure. Especially for metal particles, which are designed to exhibit toxic effects against microbes, collateral damage to healthy skin may become a limiting factor.

**Figure 4:**
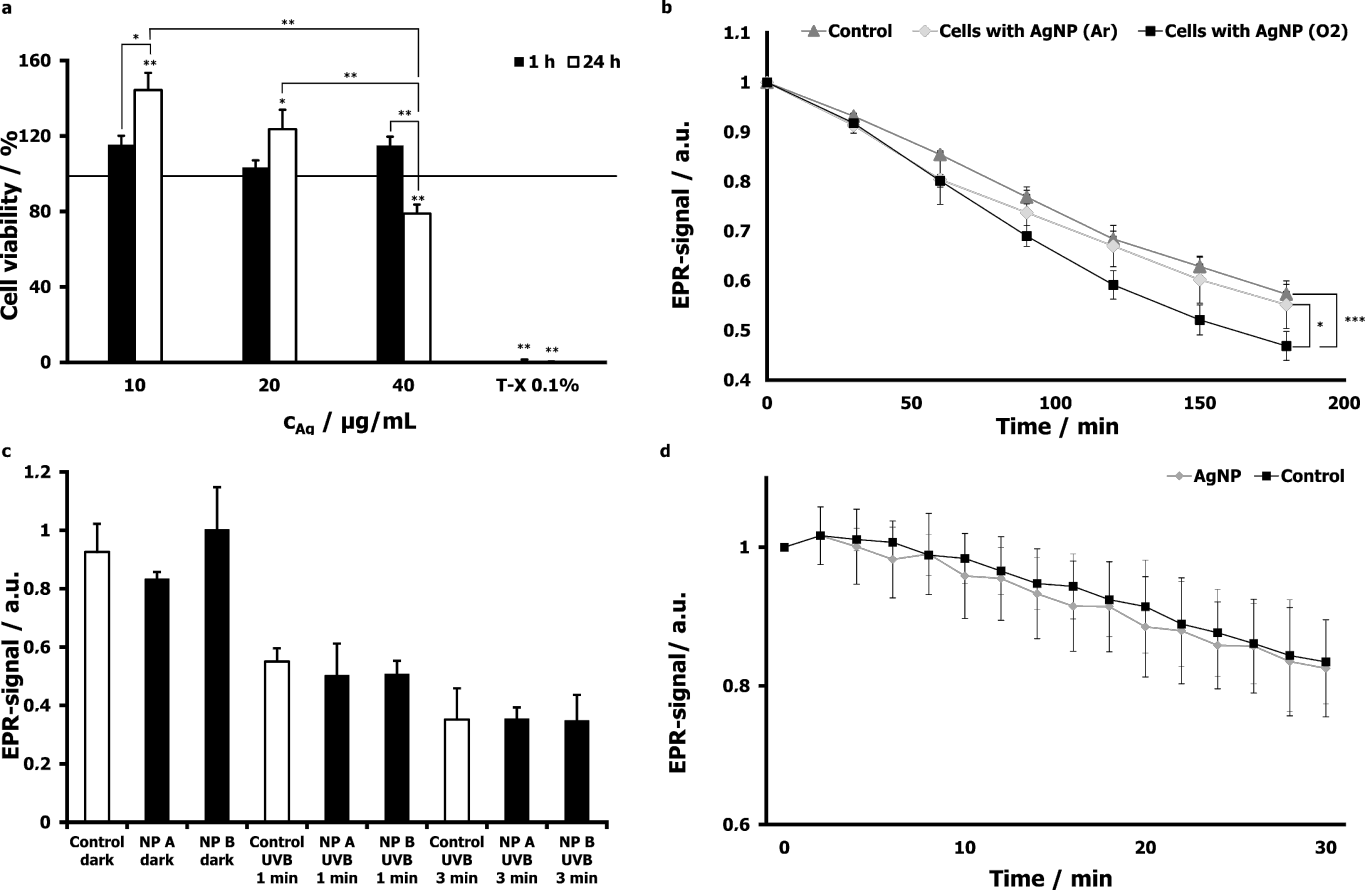
Biological responses of skin tissue and skin cells to particle exposure. The viability of HaCaT cells after 1 h and 24 h incubation with AgNP at different concentrations was assessed by using the XTT assay (a). HaCaT cells were incubated with 30 µg/mL of AgNP produced and stored under ambient air conditions or in argon atmosphere, respectively, and investigated by means of EPR spectroscopy. The used spin marker TEMPO (5 µM) becomes EPR-invisible when reacting with ROS (b). In order to analyze ROS production in whole skin, the EPR-signal intensity was monitored after the application of TiO_2_ on porcine ear samples at two different concentrations: 40 mg/mL (NPs A), 400 mg/mL (NPs B) and after irradiation after 1 or 3 min UVB light (210 and 630 mJ/cm^2^, respectively) and respective controls (c). Similarly, the EPR signal of porcine skin was followed after the topical application of AgNP (0.446 mg/mL) for 1 h. Control samples were treated with PBS only (d).

To assess the overall influence of AgNP exposure on cell viability, we investigated the influence of AgNP on cell metabolism in HaCaT cells by the XTT assay based on 2,3-bis(2-methoxy-4-nitro-5-sulfophenyl)-5-((phenylamino)carbonyl)-2*H*-tetrazolium hydroxid (Figure 4a).

Interestingly, the XTT signal increased in cells incubated with low concentrations of Ag (<20 µg/mL) but it decreased for cells incubated with 40 µg/mL. When the serum concentration in the cell culture media was reduced below the required amount of 9%, all concentrations of AgNP induced a reduction in cell viability. This finding is relevant with regard to application, because low concentrations of experimental nanoparticle preparations frequently require the addition of volumes which cause secondary dilution of cell media. Also, it could indicate that cells under suboptimal conditions are more prone to particle-induced stress. Last but not least, possible interactions between proteins in media may occur in a concentration-dependent manner and influence the particle–cell interactions, which is supported by our previous findings on the different aggregation behaviors of particles in biological media. Changes in the release of inflammatory cytokines and cell cycle alterations were correlated with those findings (unpublished data). TEM studies confirmed intracellular uptake of AgNP accumulation in vesicles, most likely endosomes (Figure 2b).

Toxicity of metal particles is widely attributed to the production of reactive oxygen species (ROS) [38] and oxidative stress. Reported studies on nanoparticle-induced oxidative stress use different read-outs for radical production including fluorochromic assays [39], depletion of antioxidants [40], enzyme activity (e.g., catalase [41], superoxide dismutase), or oxidative DNA damage. For example, reactive oxygen species-mediated DNA damage and apoptosis were detected in human skin epidermal cells after exposure to nickel nanoparticles [42]. Phototoxicity of zinc oxide nanoparticles induced the generation of oxidative DNA damage during UVA and visible light irradiation in keratinocytes [43]. Oxidative stress and skin cell toxicity were also shown for iron oxide nanoparticles [44].

In our group, we established protocols for the detection of free radicals in cells and in whole skin by electron paramagnetic resonance (EPR) spectroscopy. To detect nanoparticle-induced free radicals in cells, EPR on cell suspensions by using the spin probe TEMPO (2,2,6,6-tetramethylpiperidine-1-oxyl) was established. This semi-stable radical is a nitroxide and reacts with short-living radicals, giving the hydroxylamine which is EPR silent. The EPR signal of the spin probe decreases if radicals are present. After the addition of TEMPO to living cells, the EPR signal decreases slowly over time due to the metabolism of the cells. An irradiation of the cells with light in the UVB wavelength range induced additional radicals and decreased the EPR intensity of TEMPO faster and more drastically compared to untreated cells. When 75 nm silica particles with different surface charges were added to HaCaT cells, no oxidative stress in the dark was observed. Furthermore, irradiation with UV light showed no differences to the irradiated control cells. In contrast, uncoated TiO_2_ added to cells decreased TEMPO when irradiated with UVB (210 mJ/cm^2^), which correlates with what has been shown in literature [45]. The data were correlated with the production of IL-6. UV-radiation- and nanoparticle-induced intracellular free radical generation were measured in human keratinocytes by EPR spectroscopy [45]. This illustrated that EPR is able to measure UVB- and NP-induced ROS production in HaCaT cells. In contrast to fluorogenic assays, like the well-known dichlorofluorescein assay, which might be influenced by the light scattering properties of NP, the EPR measurement represents an alternative method to measure the oxidative effects of NP overcoming possible NP-related artefacts.

The AgNP reduced the TEMPO after 1 h incubation time in HaCaT cells through induced oxidative stress (Figure 4b). The intercellular ROS production was dependent on the formation and storage condition of the AgNP. If the AgNP were produced under ambient air conditions, more ROS were formed compared to AgNP which were produced and stored in an argon atmosphere. The oxygen in the ambient atmosphere is responsible for the formation of Ag^+^ ions by oxidation of the metallic silver nanoparticles. Silver ions are probably responsible for the induction of oxidative stress. In the argon atmosphere (in the absence of oxygen), the release of silver ions is strongly suppressed [46].

Taking the results of the uptake and cell viability into account, the data indicate that the silver ions formed during production and/or storage are mainly responsible for the induced oxidative stress and cell damage. The results are in accordance with reports in the literature, e.g., differential tolerance to AgNP depending on chloride concentrations and ionic strength and Ag^+^-induced oxidative stress in E. coli was recently demonstrated by Chambers et al. [41]. Biological responses to nanoparticle exposure on the cellular level give valuable information on possible hazardous effects of cellular particle uptake. In the whole skin, however, interactions with the skin microenvironment are much more complex. Therefore we tried to validate our in vitro findings by EPR analyses of porcine skin. Our previous investigation showed that TiO_2_ leads to ROS production of cells irradiated by UVB [45]. Thus, the same TiO_2_ particles that were used for cell experiments were investigated on porcine ear skin. While UVB irradiation alone induced high levels of free radicals detected by a marked decrease in EPR signal, no ROS could be detected after particle application alone. A lack of particle penetration with rather superficial radical production in response to UVB exposure could be an explanation. Similarly, no free radical production was detected in skin treated with silver nanoparticles (Figure 4d). Further studies are ongoing to validate this technique on whole tissue.

## Conclusion

In the presented studies, we tried to obtain a comprehensive picture of nanoparticle–skin interactions for silica, titanium dioxide and silver particles. Skin penetration studies suggest that under ex vivo conditions, the vast amount of topically applied solid nanoparticles stays on the skin surface, but deeper penetration of smaller portions, e.g., on the single particle level, is indicated by X-ray microscopy, Raman spectroscopy and flow cytometric studies on skin cells separated after skin exposure to particles. Hair follicles are important storage and putative entry sites. Penetration in biologically relevant amounts may especially occur at the site of barrier dysfunction. As a consequence, exposure of viable cells to nanoparticles may increase when particles are applied on a disturbed barrier, as it is found in patients with inflammatory skin diseases, structural defects of the barrier and open wounds or barrier dysfunction in response to excessive sunlight exposure. We show that toxic effects of particles per se have to be differentiated from secondary effects, e.g., ion release from silver particles and that cellular particle uptake and biological effects vary with experimental settings and cell type. The combination of technologies and the joint discussion of results enabled us to look at nanoparticle–skin interactions and the biological relevance of our findings from different angles.

Over the past years, the increased interest in nanoparticle interactions with biological systems has led to a strong rise in publications in the field. Gathering and processing information has become one of the biggest challenges. Our studies demonstrate the value of interdisciplinary collaborations, in this case physics, chemistry, pharmaceutical chemistry and medicine.

## Experimental

### Published work related to skin penetration and cellular uptake of amorphous silica particles

**Skin penetration of fluorescent silica particles:** Skin penetration of fluorescent silica particles was studied through fluorescence microscopy of cryosections obtained from human skin samples treated with fluorescent silica particles. The single cell suspensions were prepared after separation of epidermis from the dermis by dispase digestion. For detailed investigation of uptake by Langerhans cells, this population was enriched by magnetic cell separation (MACS) using anti-BDCA-1 (anti-CD1c) antibodies and a dendritic cell isolation kit provided by Miltenyi Biotec, Bergisch Gladbach, Germany. The procedure allows to collect highly enriched Langerhans cells from epidermis (enrichment to 75–90% compared to 2–3% in unseparated epidermis cell suspensions). Figure 1 (a,b,d,e) was modified with permission of the copyright holder from our recent publication [3]. Further details can be found in the materials and methods section of this publication.

**X-ray microscopy of gold core silica particles:** The X-ray microscopy measurements (Figure 1c) were performed on the PolLux scanning transmission STXM microscope at the Swiss Light Source, Paul Scherrer Institut, Villigen, Switzerland. The protocols for particle detection on human skin were newly developed and published in detail in our publication [4]. A detailed description of the protocols as well as larger sets of data obtained with 94 and 161 nm gold core particles with silica shells and 298 nm silica particles coated with a gold shell are available in this publication.

**Cellular uptake of functionalized silica particles and interaction with physiological media:** Data presented in Figure 3 are part of a larger study on silica particles ranging between 42 nm and 200 nm in size due to functionalization with (3-aminopropyl)triethoxysilane (APS) groups [3]. For cellular uptake studies, HaCaT cells were cultured in 75 cm^2^ cell culture flasks in RPMI supplemented with 1% penicillin/streptomycin, 2% glutamine and 10% fetal calf serum. The cells grown in an incubator with 5% CO_2_, 100% humidity at 37 °C and incubated with the different silica particles (10 μg/mL) for 2 h. Analysis was performed by using flow cytometry and confocal laser scanning microscopy. Figure 3 was modified with permission of the copyright holder from our recent publication [3]. Further details can be found in the material and method section of this publication.

### Original data related to skin penetration and biological effects of silver and titanium dioxide particles

**Raman microspectroscopy of porcine skin:** Raman microscopic measurements were performed by using the skin composition analyzer (River Diagnostics, Model 3510, Rotterdam, The Netherlands). The fingerprint region (400–2000 cm^−1^) excited by near-infrared laser radiation (785 nm, 25 mW on the skin) was used for sample analyses. Raman spectra were recorded from the skin surface down to a depth of 50 µm, in 2 µm steps. The measurement time for one spectrum was 5 s. The surface enhanced Raman scattering (SERS) signal as a result of interaction between AgNPs and the porcine skin was generated by using the same excitation conditions. The utilized Raman microscope as well as the obtained spectra were described in detail elsewhere [47–48]. Skin areas of 2 × 2 cm were incubated with 40 µL of AgNP (1.19 mg/mL). Samples were stored for 16 h in a wet chamber in the incubator (37 °C, 5% CO_2_, 100% humidity). After the incubation time, the samples were carefully cleaned with a tissue and one tape strip was done. The investigation was performed with confocal Raman microscopy. The porcine ears were obtained from freshly butchered pigs and delivered right after the slaughter. The ears were processed for the experiment within 24 h.

**Transmission electron microscopy of HaCaT Cells:** HaCaT cells were incubated for 24 h with 25 µg/mL AgNP. After fixation with 2.5% glutaraldehyde and dehydration with increasing concentrations of ethanol, cells were embedded in Epon resin. The block was sectioned (80 nm) and the cells were observed by means of a transmission electron microscope (Zeiss EM906).

**Detection of silver particle-mediated production of reactive oxygen specimen by EPR spectroscopy:** The PVP-coated silver nanoparticles were prepared as described in Loza et al. [46]. They had a negative zeta-potential of −20 mV and a diameter of the metallic core of 70 nm. They were either prepared and stored under air, leading to some degree of oxidation, or under argon [49]. To investigate the radical formation, the HaCaT cells were seeded, washed and 1∙10^6^ cells/mL were incubated with 30 µg/mL of AgNP (O_2_, *n* = 6) and (Ar, *n* = 3) and investigated by means of EPR spectroscopy as described in Ahlberg et al. [50]. The used spin marker TEMPO (5 µM) becomes EPR invisible when reacting with ROS. The EPR signal intensity of TiO_2_-treated porcine ear samples was measured with two different particles concentrations: 40 mg/mL (NPs A), 400 mg/mL (NPs B) and after 1 min or after 3 min irradiation with UVB light (210 and 630 mJ/cm^2^, respectively) and respective controls, *n* = 3. Similarly, EPR signals were assessed after 1 h of incubation of AgNP (0.446 mg/mL) on porcine skin. The skin was afterwards incubated with topically applied PCA (500 µM) for 5 min, a small sample (4.5 mm diameter) was taken and positioned in an EPR cell with a 500 µm slot. This cell was placed in the EPR spectrometer (MiniScope MS 200, Magnettech, Berlin, Germany). The settings were as follows: microwave frequency 9.4 GHz, microwave power 10 mW, central magnetic field 335 mT and sweep time 20 s. As negative control a, a skin sample with a diameter of 14 mm was incubated with PBS only. After an incubation time of 1 h, a punch biopsy of 4.5 mm in diameter was taken and placed in the EPR sprectometer.

**Assessment of cell viability through XTT Assay:** HaCaT cells were cultured in 75 cm^2^ cell culture flasks in RPMI supplemented with 1% penicillin/streptomycin, 2% glutamine and 10% fetal calf serum. The cells were grown in an incubator with 5% CO_2_, 100% humidity at 37 °C. For the XTT assay 1·10^5^ HaCaT cells/mL were seeded on a 96-well plate and incubated with the particles after 24 h [50]. Cells were washed with PBS to remove particles, which were not taken up. The XTT solution (Roche Diagnostic, Meylan, France) was prepared and added to the cells (50 µL/well). The absorbance was measured after 3 h, by using a microplate reader 2300 EnSpire (Perkin Elmer, Santa Clara, California, USA). Sample optical density (OD) was measured at wavelengths of 492 nm and 650 nm (reference wavelength).
